# Monocyte-to-high-density lipoprotein ratio as a predictor for patients with Takayasu arteritis and coronary involvement: a double-center, observational study

**DOI:** 10.3389/fimmu.2023.1120245

**Published:** 2023-06-22

**Authors:** Weiping Ci, Jin Wan, Jing Han, Kaiyuan Zou, Changjiang Ge, Lili Pan, Zening Jin

**Affiliations:** ^1^ Department of Cardiology and Macrovascular Disease, Beijing Tiantan Hospital, Capital Medical University, Beijing, China; ^2^ Department of Rheumatology and Immunology, Beijing Anzhen Hospital, Capital Medical University, Beijing Institute of Heart, Lung and Blood Vessel Diseases, Beijing, China; ^3^ Department of Rheumatology and Immunology, Beijing Tiantan Hospital, Capital Medical University, Beijing, China; ^4^ Department of Cardiology, Beijing Anzhen Hospital, Capital Medical University, Beijing Institute of Heart, Lung and Blood Vessel Diseases, Beijing, China

**Keywords:** coronary involvement, left main, monocyte-to-high-density lipoprotein ratio, prognosis, Takayasu arteritis, three-vessel disease

## Abstract

**Background:**

The implication of the monocyte-to-high-density lipoprotein ratio (MHR) in Takayasu arteritis (TAK) remains unclear.

**Objective:**

We aimed to assess the predictive value of the MHR to identify coronary involvement with TAK and determine the patient prognosis.

**Methods:**

In this retrospective study, 1,184 consecutive patients with TAK were collected and assessed, and those who were initially treated and with coronary angiography were enrolled and classified according to coronary involvement or no involvement. Binary logistic analysis was performed to assess coronary involvement risk factors. Receiver-operating characteristic analysis was used to determine the MHR value to predict coronary involvement in TAK. Major adverse cardiovascular events (MACEs) were recorded in patients with TAK and coronary involvement within a 1-year follow-up, and Kaplan–Meier survival curve analysis was conducted to compare MACEs between them stratified by the MHR.

**Results:**

A total of 115 patients with TAK were included in this study, and 41 of them had coronary involvement. A higher MHR was found for TAK with coronary involvement than for TAK without coronary involvement (*P* = 0.014). Multivariate analysis showed that the MHR is an independent risk factor for coronary involvement in TAK (odds ratio: 92.718, 95% confidence interval (*CI*): 2.813–3056.291, *P =* 0.011). With the best cut-off value of 0.35, the MHR identified coronary involvement with 53.7% sensitivity and 68.9% specificity [area under the curve (AUC): 0.639, 95% *CI*: 0.544–0.726, *P=*0.010] and identified left main disease and/or three-vessel disease (LMD/3VD) with 70.6% sensitivity and 66.3% specificity (AUC: 0.704, 95% *CI*: 0.612–0.786, *P =* 0.003) in TAK. Combined with other variables, the MHR identified coronary involvement with 63.4% sensitivity and 90.5% specificity (AUC: 0.852, 95% *CI*: 0.773–0.911, *P* < 0.001), and identified LMD/3VD with 82.4% sensitivity and 78.6% specificity (AUC: 0.827, 95% *CI*: 0.720–0.934, *P* < 0.001) in TAK. A total of 39 patients with TAK and coronary involvement were followed up for 1 year, and 5 patients suffered a MACE. Those with an MHR >0.35 had a higher MACE incidence than their counterparts with an MHR ≤0.35 (χ^2 ^=^ ^4.757, *P* = 0.029).

**Conclusions:**

The MHR could be a simple, practical biomarker for identifying coronary involvement and LMD/3VD in TAK and predicting a long-term prognosis.

## 1 Introduction

Takayasu arteritis (TAK) is a systemic vasculitis with an unknown etiology, which primarily affects the aorta and its major branches, including coronary arteries (1). Over the last decade, its prevalence was reported to be 8.4–108.3 per million in different parts of the world (2). Although this disease is rare, it is more prevalent in young Asian women (3). Coronary involvement can affect more than half of patients with TAK, causing increased mortality (4). However, approximately two-thirds of these individuals have no cardiac symptoms (5). Therefore, those patients cannot be easily identified in the early stages. Although invasive coronary angiography and non-invasive coronary computed tomography angiography (CCTA) are recommended to assess coronary artery disease (CAD), they are both time-consuming and expensive, and the former is invasive and risky (6). Several pretest probability models of CAD have been established, and most of the variables considered were age, sex, typical cardiac symptoms, and traditional cardiovascular risk factors, including high-density lipoprotein cholesterol (HDL-C); however, their utility is undermined based on meta-analysis owing to high heterogeneity in most analyses and a lack of significance based on meta-regression results (7). In fact, as a biomarker that combines monocyte counts and HDL-C, the monocyte-to-high-density lipoprotein ratio (MHR) was found to be associated with the prevalence and poor prognosis of CAD in the general population (8, 9). However, the significance of the MHR for patients with TAK and coronary involvement remains unclear. To address this issue, this study aimed to assess the predictive value of the MHR to identify coronary involvement in patients with TAK and predict their long-term prognosis.

## 2 Materials and methods

### 2.1 Patients

**Figure 1:**
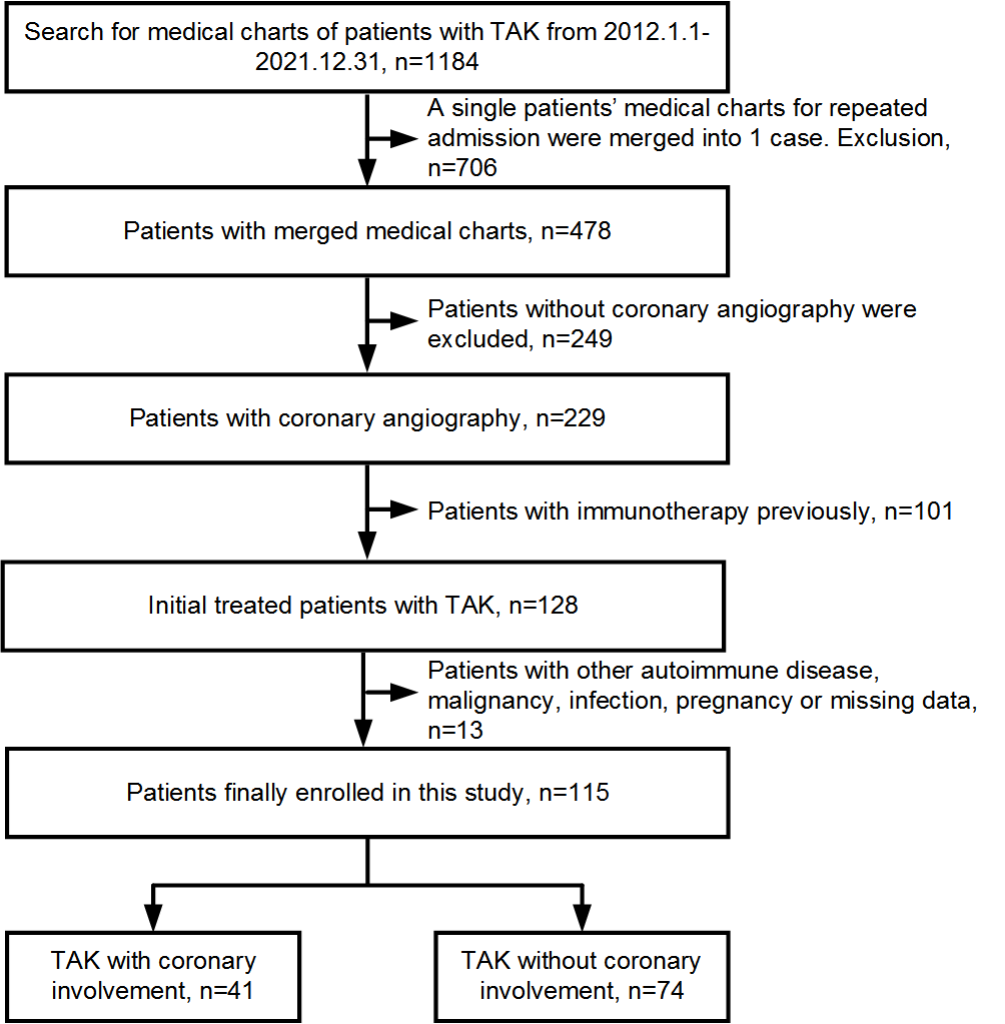
Consort flow chart of the study. TAK, Takayasu arteritis.

In this double-center, retrospective, observational study, 1,184 consecutive patients with TAK admitted to Beijing Tiantan Hospital and Beijing Anzhen Hospital between January 2012 and December 2021 were collected and assessed. TAK was diagnosed according to the classification criteria proposed by the American College of Rheumatology in 1990 (10). Finally, 115 patients who were initially treated and underwent invasive coronary angiography and/or CCTA were enrolled and grouped according to coronary involvement. Coronary involvement was defined as lesion stenosis ≥50% in any epicardial coronary artery with a diameter ≥2 mm. Patient exclusion criteria were as follows: other autoimmune diseases, malignant tumors or infections, pregnancy, and missing data based on white blood cell counts or serum lipid parameters (**Figure 1**). This study protocol was approved by the ethics committee on human research of Beijing Tiantan Hospital (No. KY2023-023-02) and Beijing Anzhen Hospital (No. 2022121X) and conformed to the ethical guidelines of the 1975 Declaration of Helsinki. Informed consent was obtained from each participant.

### 2.2 Data collection and definitions

Medical records, including demographic data, clinical characteristics, medical history, pharmacological history, laboratory findings, imaging results of the aorta and its branches, and treatment strategies at discharge, were collected. A Kerr’s score ≥2, Indian Takayasu Clinical Activity Score (ITAS) 2010 ≥2, or ITAS-A ≥5 was defined as active TAK disease (11, 12). The vascular involvement pattern of TAK was classified as Numano type I, IIa, IIb, III, IV, and V (13). Three-vessel disease (3VD) was defined as lesion stenosis ≥50% in all three major epicardial coronary arteries. The body mass index (BMI) was calculated as the weight in kilograms divided by the square of the height in meters (kg/m^2^). Hypertension was defined as blood pressure ≥140/90 mmHg with no antihypertensive drugs (14). Dyslipidemia was defined as fasting levels of triglycerides >1.7 mmol/L, total cholesterol >5.2 mmol/L, low-density lipoprotein cholesterol (LDL-C) >3.4 mmol/L, and/or HDL-C <1.0 mmol/L (15). Diabetes mellitus was defined as blood glucose levels ≥7.0 mmol/L based on fasting conditions, ≥11.1 mmol/L at 2 h post-meal or at a random time, and/or levels of glycosylated hemoglobin A1C ≥6.5% (16). The family history of premature CAD was defined as the occurrence of CAD in first-degree relatives of men <55 years or women <65 years. The estimated glomerular filtration rate (eGFR) was calculated using the following formula: 186×serum creatinine (mg/dl) ^−1.154^×age^−0.203^×0.742 (if female) (ml/min/1.73 m^2^) (17). An erythrocyte sedimentation rate (ESR) >15 mm/h for men or >20 mm/h for women indicated elevated levels. The MHR was calculated using the monocyte count divided by the HDL-C concentration. Fasting venous blood samples from each patient were drawn the next morning after admission. Total and differential white blood cells were counted using an automatic hematology analyzer (XE-2100, Sysmex, Kobe, Japan). Serum lipid profiles were collected on an automatic biochemical analyzer (AU5400, Beckman Coulter, USA). ESR data were missing for 2.4% of TAK patients with coronary involvement.

### 2.3 Follow-up

Patients with TAK and coronary involvement were followed up for 1 year. Major adverse cardiovascular events (MACEs) were defined as cardiac death, non-fatal myocardial infarction, rehospitalization due to angina or heart failure, and repeated coronary artery revascularization. For patients who revisited our hospitals after discharge, follow-up data were collected from the medical charts; for others, those were collected *via* the telephone.

### 2.4 Statistical analysis

Normally distributed numerical data were indicated as the mean ± standard deviation, and Student’s *t*-test was used for comparisons between groups. Non-normally distributed numerical data were indicated as the median and interquartile range, and a Mann–Whitney U test was used for comparisons between groups. Categorical data were indicated as frequency counts and percentages, and a Chi-square test was used for comparisons between groups. Univariate and binary logistic regression analyses were conducted to assess the coronary involvement risk factors. Variables were introduced into the multivariate analysis based on the results of the univariate analysis with a *P*-value <0.1 and clinical significance. Monocyte count and the HDL-C were not introduced into multivariate analysis together with MHR since the MHR was calculated from them. Receiver-operating characteristic (ROC) curve analysis was performed, and DeLong’s test was used to identify the best cut-off value and the predictive ability of the MHR. Kaplan–Meier survival curve analysis was applied to compare the long-term prognoses between patients stratified based on the MHR. A statistically significant difference was set as a two-sided *P*-value <0.05. IBM SPSS Statistics (version 22.0, IBM Corp., Armonk, NY, USA) and MedCalc (version 15.2.2, MedCalc Software, Ostend, Belgium) were used to perform the analyses. GraphPad Prism 7 (GraphPad Software Inc., San Diego, CA, USA) was used to generate the line art.

## 3 Results

### 3.1 Baseline characteristics of patients with Takayasu arteritis

Of the 115 patients with TAK enrolled in this study, the average age was 41.40 ± 12.27 years, and 101 (87.8%) were women. Twenty-six patients underwent invasive coronary angiography, and the other 89 underwent CCTA; 41 (35.7%) of them had coronary involvement. The age at TAK onset was older (*P* = 0.041), and the BMI was greater (*P* = 0.004) in patients with TAK and coronary involvement than in those without coronary involvement. Chest pain (*P* = 0.019) and palpitations (*P* = 0.037) were more common, whereas limb claudication (*P* = 0.030) was less common in patients with TAK and coronary involvement. A family history of premature CAD (*P* = 0.010), myocardial infarction (*P* < 0.001), and pharmacological history of β-blockers (*P* = 0.035), angiotensin-converting enzyme inhibitor or angiotensin receptor blocker (*P* = 0.003), lipid-lowering drugs (*P* = 0.027), and diuretics usage (*P* = 0.007) were more prevalent in patients with TAK and coronary involvement. Triglyceride levels (*P* < 0.001) were higher, whereas HDL-C (*P* = 0.026) and ITAS2010 (*P* = 0.043) were lower in patients with TAK and coronary involvement. A higher MHR was found for TAK with coronary involvement than for TAK without coronary involvement (*P* = 0.014). No other differences were identified in baseline characteristics between the groups (*P* > 0.05, **Table 1**).

**Table 1:** Baseline characteristics of patients with Takayasu arteritis .

Parameters	Without coronary involvement (n = 74)	With coronary involvement (n = 41)	*P-*value
Age, years	39.88 ± 11.78	44.15 ± 12.80	0.074
Male, n (%)	8 (10.8)	6 (14.6)	0.762
Age at TAK onset, years	32.15 ± 11.16	37.27 ± 13.35	**0.041**
TAK duration, months	54.00 (12.00, 144.00)	24.00 (12.00, 120.00)	0.219
BMI, kg/m^2^	22.36 ± 3.34	24.18 ± 2.88	**0.004**
Symptoms, n (%)			
Fever	4 (5.4)	1 (2.4)	0.787
Malaise	18 (24.3)	8 (19.5)	0.555
Weight loss	6 (8.1)	4 (9.8)	1.000
Arthralgia	8 (10.8)	3 (7.3)	0.780
Carotodynia	5 (6.8)	1 (2.4)	0.576
Diminished or absent pulse	11 (14.9)	5 (12.2)	0.692
Blood pressure inequality	9 (12.2)	3 (7.3)	0.620
Limb claudication	29 (39.2)	8 (19.5)	**0.030**
Chest pain	20 (27.0)	20 (48.8)	**0.019**
Dyspnea	26 (35.1)	17 (41.5)	0.502
Palpitations	5 (6.8)	9 (22.0)	**0.037**
Medical history, n (%)			
Hypertension	31 (41.9)	20 (48.8)	0.476
Dyslipidemia	20 (27.0)	16 (39.0)	0.184
Smoking	7 (9.5)	6 (14.6)	0.540
Diabetes mellitus	2 (2.7)	5 (12.2)	0.095
Family history of premature CAD	2 (2.7)	7 (17.1)	**0.010**
Myocardial infarction	0 (0.0)	9 (22.0)	**<0.001**
Heart failure	16 (21.6)	15 (36.6)	0.083
Pharmacological history, n (%)			
Antiplatelet agents	7 (9.5)	9 (22.0)	0.064
β-Blockers	6 (8.1)	9 (22.0)	**0.035**
Calcium channel blockers	13 (17.6)	10 (24.4)	0.381
ACEI/ARBs	7 (9.5)	13 (31.7)	**0.003**
Lipid-lowering drugs	8 (10.8)	11 (26.8)	**0.027**
Diuretics	3 (4.1)	9 (22.0)	**0.007**
Numano classification, n (%)			
Type I	12 (16.2)	6 (14.6)	0.823
Type IIa	5 (6.8)	2 (4.9)	1.000
Type IIb	12 (16.2)	5 (12.2)	0.561
Type III	3 (4.1)	2 (4.9)	1.000
Type IV	4 (5.4)	1 (2.4)	0.654
Type V	38 (51.4)	25 (61.0)	0.321
Active disease of TAK, n (%)	67 (90.5)	32 (78.0)	0.064
Kerr’s score	2.00 (2.00, 2.25)	2.00 (1.00, 3.00)	0.239
ITAS2010	5.00 (3.00, 8.25)	4.00 (1.00, 7.00)	**0.043**
ITAS-A	6.00 (3.00, 11.00)	6.00 (2.00, 9.00)	0.380
White blood cell, 10^9^/L	6.24 (5.34, 7.34)	6.39 (5.40, 8.12)	0.424
Neutrophil, 10^9^/L	3.85 (3.08, 5.04)	3.84 (3.30, 5.58)	0.645
Lymphocyte, 10^9^/L	1.94 ± 0.53	2.05 ± 0.63	0.348
Neutrophil-to-lymphocyte ratio	2.02 (1.77, 2.67)	2.12 (1.56, 2.69)	0.998
Monocyte, 10^9^/L	0.34 (0.28, 0.44)	0.38 (0.31, 0.47)	0.099
eGFR, ml/min/1.73 m^2^	112.70 (100.30, 130.20)	115.12 (92.52, 136.83)	0.666
Triglyceride, mmol/L	1.01 (0.69, 1.26)	1.26 (0.99, 2.16)	<**0.001**
Total cholesterol, mmol/L	4.19 ± 1.02	4.63 ± 1.31	0.052
LDL-C, mmol/L	2.52 ± 0.87	2.88 ± 1.15	0.062
HDL-C, mmol/L	1.13 (0.96, 1.44)	1.00 (0.88, 1.18)	**0.026**
Elevated ESR, n (%)	26 (35.1)	21 (52.5)	0.072
Hs-CRP, mg/L	1.99 (0.57, 10.18)	2.41 (1.07, 17.36)	0.340
MHR	0.31 (0.22, 0.39)	0.36 (0.27, 0.50)	**0.014**

Normally distributed numerical variables (e.g., age, age at TAK onset, BMI, lymphocyte, total cholesterol, and LDL-C) were indicated as the mean ± standard deviation, and Student’s t-test was used for comparisons between groups. Non-normally distributed numerical variables (e.g., TAK duration, Kerr’s score, ITAS2010, ITAS-A, white blood cell, neutrophil, neutrophil-to-lymphocyte ratio, monocyte, eGFR, triglyceride, HDL-C, hs-CRP, and MHR) were indicated as the median (P25, P75), and a Mann–Whitney U test was used for comparisons between groups. Categorical data (e.g., sex, symptoms, medical history, pharmacological history, Numano classification, active disease of TAK, and elevated ESR) were indicated as n (%), and a Chi-square test was used for comparisons between groups. ACEI, angiotensin-converting enzyme inhibitor; ARB, angiotensin receptor blocker; BMI, body mass index; CAD, coronary artery disease; eGFR, estimated glomerular filtration rate; ESR, erythrocyte sedimentation rate; HDL-C, high-density lipoprotein cholesterol; hs-CRP, high-sensitivity C-reactive protein; ITAS, the Indian Takayasu Clinical Activity Score; LDL-C, low-density lipoprotein cholesterol; MHR, monocyte-to-high-density lipoprotein ratio; TAK, Takayasu arteritis. P-values <0.05 are shown in bold.

### 3.2 Monocyte-to-high-density lipoprotein ratio for coronary involvement in Takayasu arteritis

**Figure 2:**
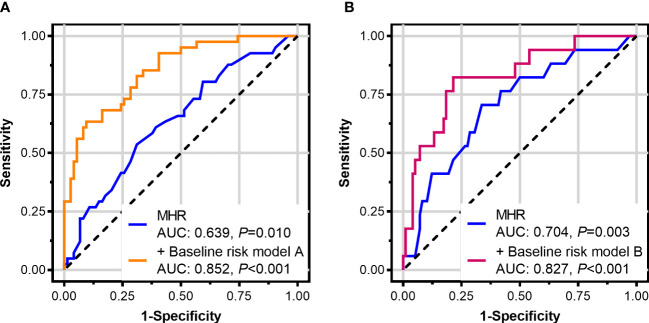
Receiver-operating characteristic curves of the monocyte-to-high-density lipoprotein ratio (MHR) in patients with Takayasu arteritis (TAK). **(A)** MHR for identifying coronary involvement and **(B)** for identifying left main disease and/or three-vessel disease (with coronary angiography as the standard). The baseline risk model A includes age, sex, age at TAK onset, body mass index, diabetes mellitus, a family history of premature coronary artery disease, lipid-lowering therapy at baseline, TAK disease activity, triglyceride, and low-density lipoprotein cholesterol. The baseline risk model B includes age, sex, body mass index, lipid-lowering therapy at baseline, triglyceride, low-density lipoprotein cholesterol, and high-sensitivity C-reactive protein.

For identifying coronary involvement risk factors in TAK, age, sex, age at TAK onset, BMI, the presence of diabetes mellitus, a family history of premature CAD, lipid-lowering therapy at baseline, TAK disease activity, triglyceride levels, LDL-C levels, and MHR were introduced into the multivariate analysis. Kerr’s score, ITAS2010, and ITAS-A were not introduced into the multivariate analysis together with TAK disease activity since the TAK disease activity was calculated from them. After adjusting for other factors, the MHR was shown to be an independent risk factor for coronary involvement in TAK [odds ratio: 92.718, 95% confidence interval (*CI*): 2.813–3056.291, *P* = 0.011, **Table 2**]. ROC curve analysis showed that the area under the curve (AUC) of the MHR for identifying coronary involvement in TAK (coronary angiography as the standard) was 0.639 (95% *CI*: 0.544–0.726, *P* = 0.010), and the best cut-off value was 0.35, with 53.7% sensitivity and 68.9% specificity. Furthermore, combined with other variables in multivariate analysis, MHR identified coronary involvement with 63.4% sensitivity and 90.5% specificity (AUC: 0.852, 95% *CI*: 0.773–0.911, *P* < 0.001) in TAK (**Figure 2A**).

**Table 2:** Risk factors for coronary involvement and left main disease and/or three-vessel disease in patients with TAK.

Parameters	For coronary involvement						For LMD/3VD					
	Univariate analysis			Multivariate analysis			Univariate analysis			Multivariate analysis		
	OR	95% *CI*	*P-*value	OR	95% *CI*	*P-*value	OR	95% *CI*	*P-*value	OR	95% *CI*	*P-*value
Age	1.030	0.997–1.065	0.076	0.983	0.920–1.049	0.601	1.019	0.975–1.064	0.401	1.013	0.957–1.073	0.648
Sex	0.707	0.227–2.200	0.549	1.864	0.352–9.867	0.464	0.590	0.146–2.383	0.459	0.773	0.146–4.081	0.761
Age at TAK onset	1.036	1.003–1.071	**0.033**	1.032	0.968–1.101	0.332	1.027	0.984–1.072	0.217			
BMI	1.199	1.054–1.363	**0.006**	1.098	0.937–1.286	0.250	1.257	1.051–1.504	**0.012**	1.238	0.993–1.544	0.058
Diabetes mellitus	5.000	0.925–27.041	0.062	5.386	0.717–40.439	0.102	0.958	0.108–8.499	0.970			
Family history of premature CAD	7.412	1.462–37.585	**0.016**	6.688	1.015–44.071	**0.048**	1.733	0.328–9.150	0.517			
Lipid-lowering therapy at baseline	3.025	1.104–8.287	**0.031**	4.368	1.150–16.592	**0.030**	2.500	0.763–8.192	0.130	3.665	0.856–15.696	0.080
Active disease of TAK	0.371	0.127–1.087	0.071	0.189	0.044–0.819	**0.026**	0.714	0.180–2.828	0.631			
Monocyte	6.438	0.374–110.839	0.200				5.523	0.157–194.145	0.347			
Triglyceride	3.702	1.804–7.597	**<0.001**	2.955	1.333–6.550	**0.008**	1.953	1.100–3.468	**0.022**	1.559	0.762–3.189	0.224
LDL-C	1.446	0.974–2.147	0.067	1.618	0.911–2.873	0.101	1.572	0.966–2.559	0.069	1.438	0.795–2.600	0.230
HDL-C	0.364	0.104–1.276	0.114				0.022	0.002–0.318	**0.005**			
Hs-CRP	1.000	0.982–1.019	0.984				1.009	0.989–1.030	0.363	1.021	0.995–1.048	0.109
MHR	21.612	1.662–281.116	**0.019**	92.718	2.813–3056.291	**0.011**	60.208	2.855–1269.687	**0.008**	52.130	1.027–2645.651	**0.048**

BMI, body mass index; CAD, coronary artery disease; CI, confidence interval; HDL-C, high-density lipoprotein cholesterol; hs-CRP, high-sensitivity C-reactive protein; LDL-C, low-density lipoprotein cholesterol; LMD/3VD, left main disease and/or three-vessel disease; MHR, monocyte-to-high-density lipoprotein ratio; OR, odds ratio; TAK, Takayasu arteritis. P-values <0.05 are shown in bold.

### 3.3 Monocyte-to-high-density lipoprotein ratio for left main disease and/or three-vessel disease with Takayasu arteritis

Among the 115 patients with TAK, 17 (14.8%) had left main disease and/or three-vessel disease (LMD/3VD; specifically, 11 patients with LMD, 3 with 3VD, and 3 with LMD and 3VD). The BMI was greater (*P* = 0.009); chest pain (*P* = 0.024) and myocardial infarction (*P* = 0.002) were more prevalent in patients with TAK and LMD/3VD than in those without LMD/3VD. Levels of triglyceride (*P* = 0.021) and high-sensitivity C-reactive protein (*P* = 0.022) were higher, whereas HDL-C levels (*P* = 0.001) were lower in patients with TAK and LMD/3VD. A higher MHR was found for TAK with LMD/3VD than for TAK without LMD/3VD (*P* = 0.007). No other differences were identified in baseline characteristics between the groups (*P* > 0.05, **Supplementary Table 1**).

**Figure 3:**
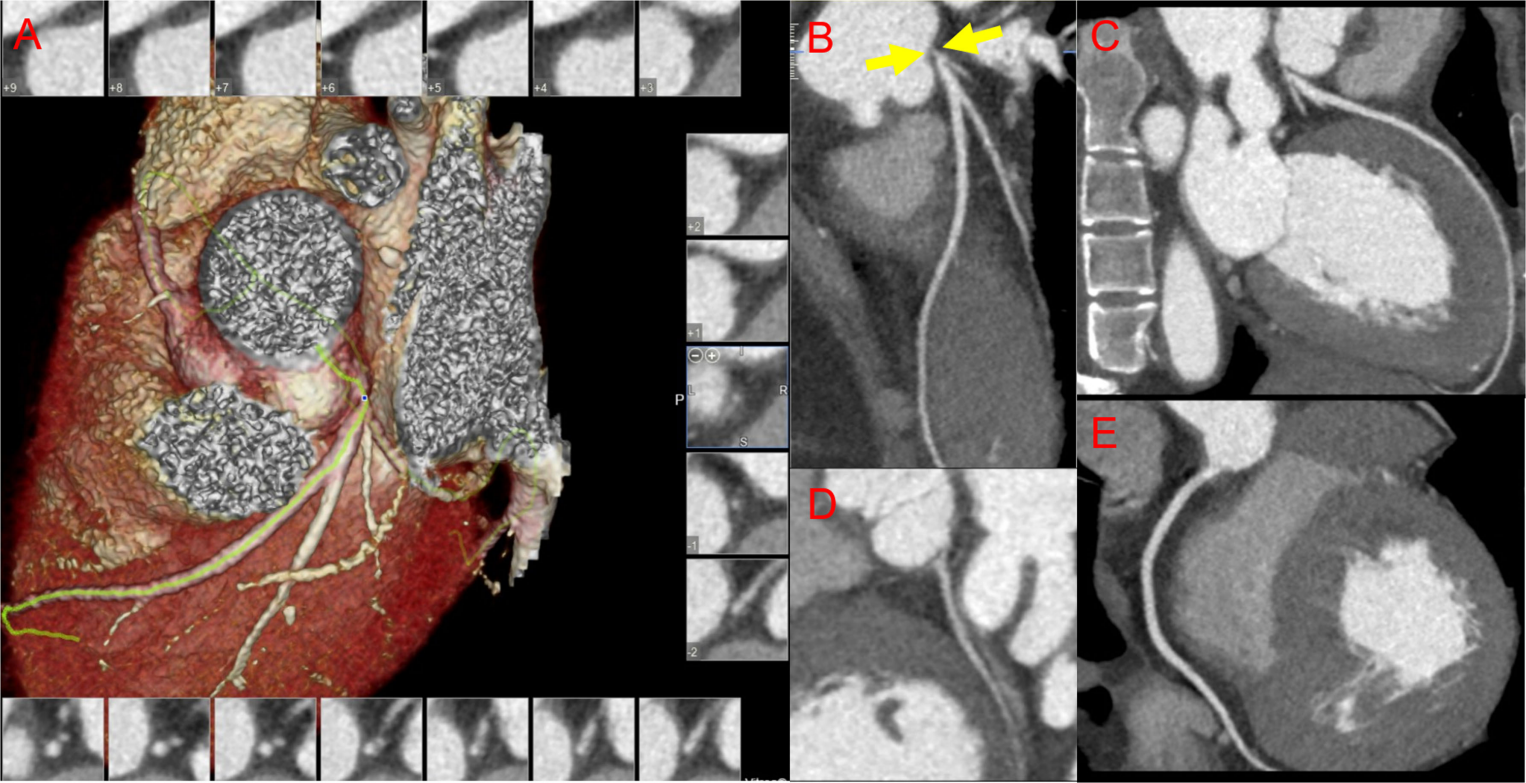
Images for coronary involvement in Takayasu arteritis. A 24-year-old woman with newly diagnosed Takayasu arteritis complicated with old myocardial infarction and heart failure presented with dyspnea for 1 year and had an monocyte-to-high-density lipoprotein ratio of 0.66. **(A)** Reconstructed imaging of coronary computed tomography angiography (CCTA); CCTA demonstrates severe stenosis in the left main coronary artery **(B)** (yellow arrows), and normal vessels of the left anterior descending coronary artery **(C)**, the left circumflex coronary artery **(D)**, and the right coronary artery **(E)**.

For identifying LMD/3VD risk factors in TAK, age, sex, BMI, lipid-lowering therapy at baseline, triglyceride levels, LDL-C levels, high-sensitivity C-reactive protein levels, and MHR were introduced into the multivariate analysis. After adjusting for other factors, the MHR was shown to be an independent risk factor for LMD/3VD in TAK (odds ratio: 52.130, 95% *CI*: 1.027–2,645.651, *P*=0.048, **Table 2**). ROC curve analysis showed that the AUC of the MHR for identifying LMD/3VD in patients with TAK (coronary angiography as the standard) was 0.704 (95% *CI*: 0.612–0.786, *P* = 0.003), and the best cut-off value was 0.35, with 70.6% sensitivity and 66.3% specificity. Furthermore, combined with other variables in multivariate analysis, the MHR identified LMD/3VD with 82.4% sensitivity and 78.6% specificity (AUC: 0.827, 95% *CI*: 0.720–0.934, *P* < 0.001) in TAK (**Figure 2B**). **Figure 3** shows images for LMD in a patient with TAK with an MHR of 0.66.

### 3.4 Monocyte-to-high-density lipoprotein ratio for major adverse cardiovascular events in Takayasu arteritis with coronary involvement

Among 41 patients with TAK and coronary involvement, 39 (95.1%) were followed up for 365.00 (365.00, 365.00) days, and 5 (5/39, 12.8%) suffered a MACE. As shown in **Table 3**, the monocyte count was higher (*P* = 0.001), and HDL-C levels were lower (*P* < 0.001) in patients with a high MHR (MHR >0.35, 21 cases) than in patients with a low MHR (MHR ≤0.35, 18 cases). There were 11 patients with a high MHR who underwent revascularization [4 patients with percutaneous coronary intervention (PCI), 6 patients with coronary artery bypass grafting (CABG), and 1 patient with both PCI and CABG], while none with a low MHR underwent revascularization. CABG was more prevalent in patients with a high MHR (*P* = 0.010). In addition, 5 of the 11 patients who underwent coronary artery revascularization did not start immunosuppressive therapy at discharge.

**Table 3:** Clinical characteristics, laboratory findings, and treatment strategies of follow-up patients with TAK and coronary involvement.

Parameters	MHR ≤0.35 (n = 18)	MHR >0.35 (n = 21)	*P-*value
Age, years	45.44 ± 12.24	44.00 ± 13.49	0.730
Male, n (%)	1 (5.6)	5 (23.8)	0.190
Age at TAK onset, years	39.00 (29.75, 45.00)	37.00 (25.50, 51.50)	0.666
TAK duration, months	24.00 (12.00, 120.00)	24.00 (12.00, 96.00)	0.813
BMI, kg/m^2^	23.52 ± 3.04	25.06 ± 2.52	0.094
Symptoms, n (%)			
Fever	1 (5.6)	0 (0.0)	0.462
Malaise	4 (22.2)	3 (14.3)	0.682
Weight loss	0 (0.0)	3 (14.3)	0.235
Arthralgia	3 (16.7)	0 (0.0)	0.089
Carotodynia	1 (5.6)	0 (0.0)	0.462
Diminished or absent pulse	3 (16.7)	2 (9.5)	0.647
Blood pressure inequality	1 (5.6)	2 (9.5)	1.000
Limb claudication	4 (22.2)	4 (19.0)	1.000
Chest pain	5 (27.8)	13 (61.9)	0.054
Dyspnea	5 (27.8)	11 (52.4)	0.192
Palpitations	5 (27.8)	3 (14.3)	0.432
Medical history, n (%)			
Hypertension	6 (33.3)	14 (66.7)	0.056
Dyslipidemia	8 (44.4)	8 (38.1)	0.752
Smoking	2 (11.1)	4 (19.0)	0.667
Diabetes mellitus	3 (16.7)	2 (9.5)	0.647
Family history of premature CAD	3 (16.7)	4 (19.0)	1.000
Myocardial infarction	2 (11.1)	7 (33.3)	0.139
Heart failure	3 (16.7)	10 (47.6)	0.051
Numano classification, n (%)			
Type I	3 (16.7)	3 (14.3)	1.000
Type IIa	1 (5.6)	1 (4.8)	1.000
Type IIb	3 (16.7)	2 (9.5)	0.647
Type III	1 (5.6)	1 (4.8)	1.000
Type IV	0 (0.0)	1 (4.8)	1.000
Type V	10 (55.6)	13 (61.9)	0.752
Active disease of TAK, n (%)	12 (66.7)	18 (85.7)	0.255
White blood cell, 10^9^/L	6.76 ± 2.05	6.95 ± 1.92	0.768
Neutrophil, 10^9^/L	3.77 (2.82, 5.38)	3.93 (3.48, 5.75)	0.490
Lymphocyte, 10^9^/L	2.07 ± 0.72	2.08 ± 0.55	0.993
Neutrophil-to-lymphocyte ratio	2.10 (1.48, 2.67)	2.09 (1.57, 2.62)	0.602
Monocyte, 10^9^/L	0.33 ± 0.08	0.44 ± 0.10	**0.001**
eGFR, mL/min/1.73 m^2^	116.17 ± 33.70	115.00 ± 28.74	0.907
Triglyceride, mmol/L	1.20 (0.97, 1.95)	1.67 (1.03, 2.39)	0.269
Total cholesterol, mmol/L	4.77 ± 1.30	4.53 ± 1.42	0.595
LDL-C, mmol/L	2.87 ± 1.14	2.90 ± 1.24	0.923
HDL-C, mmol/L	1.19 (1.05, 1.42)	0.92 (0.82, 1.00)	**<0.001**
Elevated ESR, n (%)	8 (44.4)	11 (55.0)	0.746
Hs-CRP, mg/L	2.08 (0.45, 16.92)	2.97 (1.34, 8.03)	0.364
Treatment at discharge, n (%)			
Glucocorticoids	8 (44.4)	10 (47.6)	1.000
Immunosuppressants	10 (55.6)	11 (52.4)	1.000
Immunosuppressive therapy	10 (55.6)	14 (66.7)	0.477
Antiplatelet agents	15 (83.3)	18 (85.7)	1.000
β-blockers	7 (38.9)	14 (66.7)	0.113
Calcium channel blockers	4 (22.2)	6 (28.6)	0.726
ACEI/ARBs	5 (27.8)	11 (52.4)	0.192
Statins	15 (83.3)	16 (76.2)	0.702
Diuretics	3 (16.7)	5 (23.8)	0.702
Percutaneous coronary intervention	0 (0.0)	5 (23.8)	0.050
Coronary artery bypass grafting	0 (0.0)	7 (33.3)	**0.010**
Follow-up time, days	365.00 (365.00, 365.00)	365.00 (362.00, 365.00)	0.213

Normally distributed numerical variables (e.g., age, BMI, white blood cell, lymphocyte, monocyte, eGFR, total cholesterol, and LDL-C) were indicated as the mean ± standard deviation, and a Student’s t-test was used for comparisons between groups. Non-normally distributed numerical variables (e.g., age at TAK onset, TAK duration, neutrophil, neutrophil-to-lymphocyte ratio, triglyceride, HDL-C, hs-CRP, and follow-up time) were indicated as the median (P25, P75), and a Mann–Whitney U test was used for comparisons between groups. Categorical data (e.g., sex, symptoms, medical history, Numano classification, active disease of TAK, elevated ESR, and treatment at discharge) were indicated as n (%), and a Chi-square test was used for comparisons between groups. ACEI, angiotensin-converting enzyme inhibitor; ARB, angiotensin receptor blocker; BMI, body mass index; CAD, coronary artery disease; eGFR, estimated glomerular filtration rate; ESR, erythrocyte sedimentation rate; HDL-C, high-density lipoprotein cholesterol; hs-CRP, high-sensitivity C-reactive protein; LDL-C, low-density lipoprotein cholesterol; MHR, monocyte-to-high-density lipoprotein ratio; TAK, Takayasu arteritis. P-value <0.05 are shown in bold.

**Figure 4:**
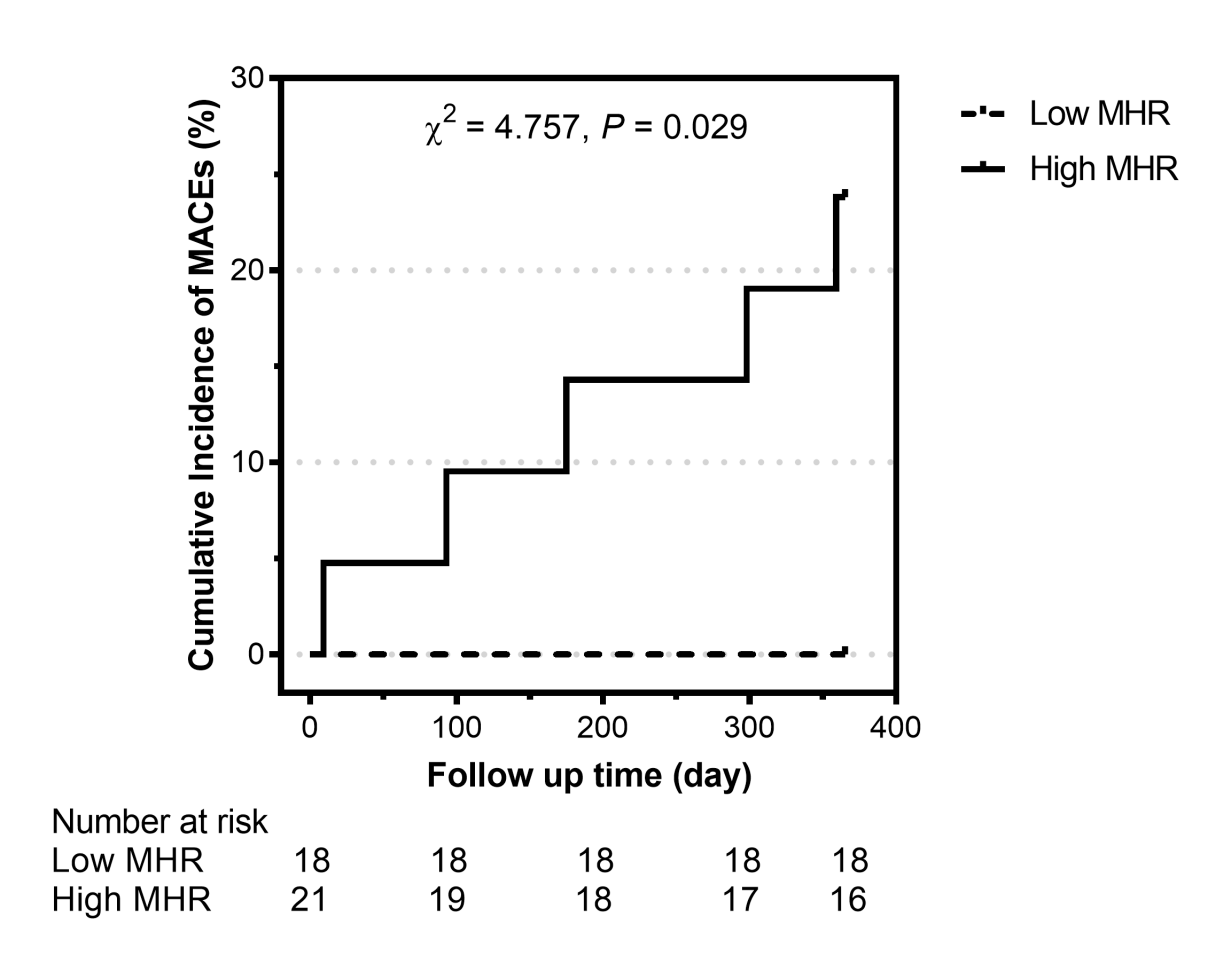
Cumulative incidence of major adverse cardiovascular events (MACEs) in patients with Takayasu arteritis and coronary involvement stratified by the monocyte-to-high-density lipoprotein ratio (MHR). Low MHR: MHR ≤0.35; high MHR: MHR >0.35.

During the 1-year follow-up, five patients with a high MHR (5/21, 23.8%) suffered a MACE (one man who underwent CABG and received no immunosuppressive therapy died of acute myocardial infarction on day 9, one man with methotrexate and tocilizumab usage rehospitalized owing to angina and underwent PCI at day 93, one man who underwent PCI with stent implantation and received no immunosuppressive therapy suffered a non-fatal myocardial infarction at day 175, one woman who underwent CABG and received glucocorticoids (0.5mg/kg/d) in combination with methotrexate and tocilizumab rehospitalized owing to angina and heart failure at day 298, and one woman with tocilizumab usage rehospitalized owing to heart failure at day 359, after discharge). However, no patients with a low MHR suffered a MACE. Kaplan–Meier survival curve analysis showed that patients with a high MHR had a higher MACE rate than those with a low MHR (χ^2 ^=^ ^4.757, *P* = 0.029; **Figure 4**).

## 4 Discussion

This study explored the value of the MHR for patients with TAK and coronary involvement. Our findings showed that it is an independent risk factor for coronary involvement with TAK and could be used to identify coronary involvement and LMD/3VD in TAK. Further, during the 1-year follow-up period, patients with a high MHR had a higher MACE rate than those with a low MHR in TAK with coronary involvement.

As compared with the general population, a higher prevalence of subclinical atherosclerosis and cardiovascular events in patients with autoimmune diseases such as rheumatoid arthritis, systemic lupus erythematosus, and systemic vasculitis including TAK has been extensively documented (18–20). However, the mechanism underlying cardiovascular events relevant to autoimmune diseases has not been completely elucidated. It seems to occur as a result of the accelerated atherosclerosis caused by prolonged endothelial activation in a proinflammatory environment and a prothrombotic and procoagulant state, combined with traditional cardiovascular risk factors (21, 22). In fact, as a well-established traditional cardiovascular risk factor, dyslipidemia can accelerate both inflammation and atherosclerosis in these conditions, while oxidized low-density lipoprotein is now recognized as a bridge between autoimmunity and atherosclerosis (23, 24). However, Giles et al. (25) found that patients with rheumatic arthritis have a heavier atherosclerotic burden in their coronary arteries, but their serum HDL-C concentrations are significantly higher compared with healthy controls. It indicates that a lipid paradox should been considered with rheumatic diseases. Kang et al. (5) reported coronary artery abnormalities in 53.2% of patients with TAK based on CCTA, whereas 66.1% of these patients did not have cardiac symptoms. As coronary angiography is relatively complex and tends to be performed on patients with cardiac symptoms in clinical practice, a simple laboratory screening test for coronary involvement in TAK would be of great value to mitigate missed diagnoses. Monocytes are involved in the inflammatory responses and contribute to all stages of atherosclerosis, whereas HDL-C can suppress the pro-oxidative and proinflammatory effects of monocytes by inhibiting the activation of CD11b integrin (26, 27). As a laboratory biomarker calculated from peripheral white blood cell differential counts and serum lipid levels, the MHR can be easily obtained. Recently, the MHR was reported to be an indicator related to the prevalence of CAD in the general population (8). We hypothesized that the MHR, an indicator combining monocyte counts with HDL-C levels, may be also a promising biomarker for predicting coronary involvement in TAK. Consistent with this, we found the MHR was significantly higher in patients with TAK and coronary involvement compared with those without coronary involvement. However, this difference may be underestimated. In this study, there was a 16.0% higher prevalence of lipid-lowering drug usage in patients with coronary involvement than in those without coronary involvement at baseline. Statin usage, as a dominant lipid-lowering therapy, has been recommended to be a significant primary or secondary prevention strategy of CAD, which might mildly increase HDL-C levels and decrease the MHR correspondingly (15).

Both traditional factors (such as age) and disease-related factors (such as Numano type V) have been found to be related to coronary involvement in TAK (4, 28). In the present study, ITAS2010 values increased in patients without coronary involvement compared to those with coronary involvement; however, no significant differences were detected in Kerr’s score or ITAS-A values. Although the three assessment tools are all designed to quantify disease activity for TAK, they have some differences in items. For example, different from the other two disease activity scores, ITAS2010 includes only clinical findings and physical examination items. Nevertheless, acute-phase reactants are not included in ITAS2010, which might explain the inconsistency of findings in assessing TAK disease activity between ITAS2010 and Kerr’s score or ITAS-A values. In multivariate analysis, non-active disease was found to be a risk factor for coronary involvement in TAK. It indicates that the vessel damage secondary to vasculitis might be more prominent than coronary arteritis in coronary involvement with TAK. Consistent with that, premature atherosclerosis as well as increased arterial stiffness occurred independently from atherosclerosis have been observed in TAK (29). Interestingly, a negative correlation was found for the arterial stiffness with peripheral neutrophil-to-lymphocyte ratio, neutrophil counts, and white blood cell counts, whereas a positive correlation was found for that with lymphocyte counts in patients with active TAK (30). However, as no significant differences in these peripheral blood cell parameters were detected between groups, there might be a similar arterial stiffness in TAK patients with coronary involvement and those without coronary involvement. In the univariate analysis, the MHR rather than HDL-C level was found to be a risk factor for coronary involvement in TAK. It was also true for the MHR after adjusting for other traditional cardiovascular risk factors, disease-related factors, and lipid-lowering therapy at baseline. Specifically, each 1 unit increase in the MHR was associated with a 92.7-fold increased risk of coronary involvement. In addition, our results confirmed that the MHR is a valuable biomarker for predicting coronary involvement in TAK. Furthermore, our results showed that the prevalence of LMD/3VD in patients with TAK and coronary involvement (41.5%) was higher than that reported in the general population in previous studies (20%–36%) (31). This indicates that not only atherosclerosis but also vasculitis contribute to coronary stenosis in TAK (32). LMD/3VD is associated with high mortality and adverse events in CAD (31). However, CABG has been demonstrated to provide significantly better long-term outcomes than PCI for patients with 3VD (33). Therefore, the early identification of LMD/3VD would be of great significance in determining optimal treatment strategies. Our results showed that each 1 unit increase in the MHR was associated with a 52.1-fold increased risk of LMD/3VD in patients with TAK. The MHR, as a simple and practical biomarker, could serve as an indicator for identifying LMD/3VD in patients with TAK. Notably, combined with other variables, the MHR could get better sensitivity and specificity for identifying coronary involvement or LMD/3VD.

A MACE has been found in 12.8% of patients with TAK and coronary involvement during 1-year follow-up in our study. Meta-analysis showed that the MHR is associated with MACEs in patients with acute coronary syndrome (27). However, to the best of our knowledge, the prognostic utility of the MHR for patients with autoimmune diseases and coronary involvement has not been explored. To further verify the value of MHR in predicting the long-term prognosis in TAK with coronary involvement, we compare the 1-year MACEs between patients stratified by the best cut-off value with an MHR of 0.35 for coronary involvement. Interestingly, our results showed that all of the five patients suffered 1-year MACEs had a high MHR at baseline, and patients with an MHR >0.35 had a 23.8% higher prevalence of MACEs than those with an MHR ≤0.35. It indicates that patients with a high MHR also should be paid more attention to MACE occurrence than those with a low MHR in TAK and coronary involvement. Furthermore, several other clinical factors such as active disease at baseline and male sex, as well as PCI, have been demonstrated to be associated with a worse long-term prognosis for TAK with coronary stenosis (34, 35). Similar to that, there was a nearly 44% higher prevalence of a 1-year MACE occurrence in male patients with TAK and coronary involvement than in their female counterparts. In terms of those with TAK underwent coronary artery revascularization, we found that two of the five patients with no immunosuppressive therapy suffered a MACE within 6 months after discharge, compared to only one of the other six patients with immunosuppressive therapy suffered a MACE nearly 10 months after discharge. Similarly, Liu et al. (36) reported that all the seven patients with isolated coronary arteritis who underwent revascularization and did not receive immunosuppressive therapy suffered restenosis within 6 months. However, long-term stability had been obtained after receiving systemic glucocorticoids in combination with immunosuppressants, especially cyclophosphamide, appropriately. Therefore, for patients with TAK and coronary involvement, especially those with an MHR >0.35, optimal disease control might reduce the MACE incidence and improve their long-term outcomes. Moreover, individual exercise prescription may be helpful to the rehabilitation of cardiovascular disease for patients with TAK (37).

The present study had several limitations. Although it was a double-center study, a selection bias may have existed owing to its retrospective and observational design. In addition, we did not exclude patients who were taking lipid-lowering therapy at baseline, in order to reduce selection bias. In addition, although our results were clear, TAK is a rare disorder, and the sample size of patients with TAK and coronary involvement was relatively small. Our findings should be confirmed by future multicenter, large-sized prospective studies.

## 5 Conclusions

Our study demonstrates that the MHR could be a simple and practical biomarker for identifying coronary involvement and LMD/3VD in TAK and for predicting the patient prognosis. Patients with a high MHR should be paid more attention to coronary involvement and MACE occurrence than those with a low MHR in TAK.

## Data availability statement

The raw data supporting the conclusions of this article will be made available by the authors, without undue reservation.

## Ethics statement

The studies involving human participants were reviewed and approved by the ethics committee on human research of Beijing Tiantan Hospital (No. KY2023-023-02) and Beijing Anzhen Hospital (No. 2022121X). Written informed consent for participation was not required for this study in accordance with the national legislation and the institutional requirements.

## Author contributions

WC conceived and designed the study, collected the data, performed the statistical analyses, and drafted the manuscript. JW, JH and KZ helped to collect the data and revise the manuscript. CG participated in the design of the study and helped to revise the manuscript. LP and ZJ directed the concept and design, revised the manuscript, and were responsible for the study. All authors contributed to the article and approved the submitted version.

## References

[1] Yuan SM Lin HZ. Coronary artery involvements in takayasu arteritis: systematic review of reports. Gen Thorac Cardiovasc Surg (2020) 68:883–904. doi: 10.1007/s11748-020-01378-3 32430746

[2] Watts RA Hatemi G Burns JC Mohammad AJ. Global epidemiology of vasculitis. Nat Rev Rheumatol (2022) 18:22–34. doi: 10.1038/s41584-021-00718-8 34853411 PMC8633913

[3] Rutter M Bowley J Lanyon PC Grainge MJ Pearce FA. A systematic review and meta-analysis of the incidence rate of takayasu arteritis. Rheumatology (2021) 60:4982–90. doi: 10.1093/rheumatology/keab406 PMC8566298 33944899

[4] Wang H Liu Z Shen Z Fang L Zhang S. Impact of coronary involvement on long-term outcomes in patients with takayasu's arteritis. Clin Exp Rheumatol (2020) 38:1118–26. 32083549

[5] Kang EJ Kim SM Choe YH Lee GY Lee KN Kim DK. Takayasu arteritis: assessment of coronary arterial abnormalities with 128-section dual-source CT angiography of the coronary arteries and aorta. Radiology (2014) 270:74–81. doi: 10.1148/radiol.13122195 24009351

[6] Barone-Rochette G Bruere D Mansencal N. How to explore coronary artery disease? Arch Cardiovasc Dis (2019) 112:546–9. doi: 10.1016/j.acvd.2019.05.002 31331760

[7] Mincarone P Bodini A Tumolo MR Vozzi F Rocchiccioli S Pelosi G. Discrimination capability of pretest probability of stable coronary artery disease: a systematic review and meta-analysis suggesting how to improve validation procedures. BMJ Open (2021) 11:e047677. doi: 10.1136/bmjopen-2020-047677 PMC8268916 34244268

[8] Zhang M Wu S Xu S Chen S. Impact of monocyte to high-density lipoprotein ratio on the identification of prevalent coronary heart disease: insights from a general population. Postgraduate Med (2021) 133:822–9. doi: 10.1080/00325481.2021.1957265 34281466

[9] Sun M Zhao D Zhang Y Zhai Y Ye M Wang X. Prognostic utility of monocyte to high-density lipoprotein ratio in patients with acute coronary syndrome: a meta-analysis. Am J Med Sci (2020) 359:281–6. doi: 10.1016/j.amjms.2020.01.018 32245567

[10] Arend WP Michel BA Bloch DA Hunder GG Calabrese LH Edworthy SM. The American college of rheumatology 1990 criteria for the classification of takayasu arteritis. Arthritis Rheumatism (1990) 33:1129–34. doi: 10.1002/art.1780330811 1975175

[11] Kerr GS Hallahan CW Giordano J Leavitt RY Fauci AS Rottem M. Takayasu arteritis. Ann Internal Med (1994) 120:919–29. doi: 10.7326/0003-4819-120-11-199406010-00004 7909656

[12] Misra R Danda D Rajappa SM Ghosh A Gupta R Mahendranath KM. Development and initial validation of the Indian takayasu clinical activity score (ITAS2010). Rheumatology (2013) 52:1795–801. doi: 10.1093/rheumatology/ket128 23594468

[13] Hata A Noda M Moriwaki R Numano F. Angiographic findings of takayasu arteritis: new classification. Int J Cardiol (1996) 54 Suppl:S155–63. doi: 10.1016/s0167-5273(96)02813-6 9119518

[14] Jones NR McCormack T Constanti M McManus RJ. Diagnosis and management of hypertension in adults: NICE guideline update 2019. Br J Gen Pract J R Coll Gen Practitioners (2020) 70:90–1. doi: 10.3399/bjgp20X708053 PMC7018407 32001477

[15] Joint Committee for Guideline Revision. 2016 Chinese guidelines for the management of dyslipidemia in adults. J Geriatric Cardiol JGC (2018) 15:1–29. doi: 10.11909/j.issn.1671-5411.2018.01.011 PMC5803534 29434622

[16] Lian F Ni Q Shen Y Yang S Piao C Wang J. International traditional Chinese medicine guideline for diagnostic and treatment principles of diabetes. Ann Palliative Med (2020) 9:2237–50. doi: 10.21037/apm-19-271 32648463

[17] Soares AA Eyff TF Campani RB Ritter L Camargo JL Silveiro SP. Glomerular filtration rate measurement and prediction equations. Clin Chem Lab Med (2009) 47:1023–32. doi: 10.1515/CCLM.2009.263 19728843

[18] Ruscitti P Margiotta DPE Macaluso F Iacono D D'Onofrio F Emmi G. Subclinical atherosclerosis and history of cardiovascular events in Italian patients with rheumatoid arthritis: results from a cross-sectional, multicenter GIRRCS (Gruppo italiano di ricerca in reumatologia clinica e sperimentale) study. Med (Baltimore) (2017) 96:e8180. doi: 10.1097/MD.0000000000008180 PMC5662366 29049200

[19] Leone P Prete M Malerba E Bray A Susca N Ingravallo G. Lupus vasculitis: an overview. Biomedicines (2021) 9:1626. doi: 10.3390/biomedicines9111626 34829857 PMC8615745

[20] Clifford AH Cohen Tervaert JW. Cardiovascular events and the role of accelerated atherosclerosis in systemic vasculitis. Atherosclerosis (2021) 325:8–15. doi: 10.1016/j.atherosclerosis.2021.03.032 33873090

[21] Atzeni F Nucera V Gerratana E Fiorenza A Gianturco L Corda M. Cardiovascular consequences of autoimmune rheumatic diseases. Curr Vasc Pharmacol (2020) 18:566–79. doi: 10.2174/1570161118666200127142936 31985379

[22] Numano F Kishi Y Tanaka A Ohkawara M Kakuta T Kobayashi Y. Inflammation and atherosclerosis. atherosclerotic lesions in takayasu arteritis. Ann N Y Acad Sci (2000) 902:65–76. doi: 10.1111/j.1749-6632.2000.tb06301.x 10865826

[23] Wang Y Yu H He J. Role of dyslipidemia in accelerating inflammation, autoimmunity, and atherosclerosis in systemic lupus erythematosus and other autoimmune diseases. Discovery Med (2020) 30:49–56. 33357362

[24] Suciu CF Prete M Ruscitti P Favoino E Giacomelli R Perosa F. Oxidized low density lipoproteins: the bridge between atherosclerosis and autoimmunity. possible implications in accelerated atherosclerosis and for immune intervention in autoimmune rheumatic disorders. Autoimmun Rev (2018) 17:366–75. doi: 10.1016/j.autrev.2017.11.028 29425936

[25] Giles JT Wasko MCM Chung CP Szklo M Blumenthal RS Kao A. Exploring the lipid paradox theory in rheumatoid arthritis: associations of low circulating low-density lipoprotein concentration with subclinical coronary atherosclerosis. Arthritis Rheumatol (2019) 71:1426–36. doi: 10.1002/art.40889 PMC6716986 30883031

[26] Ganjali S Gotto AM Jr. Ruscica M Atkin SL Butler AE Banach M. Monocyte-to-HDL-cholesterol ratio as a prognostic marker in cardiovascular diseases. J Cell Physiol (2018) 233:9237–46. doi: 10.1002/jcp.27028 30076716

[27] Haybar H Pezeshki SMS Saki N. Evaluation of complete blood count parameters in cardiovascular diseases: an early indicator of prognosis? Exp Mol Pathol (2019) 110:104267. doi: 10.1016/j.yexmp.2019.104267 31194963

[28] Li T Du J Gao N Guo X Pan L. Numano type V takayasu arteritis patients are more prone to have coronary artery involvement. Clin Rheumatol (2020) 39:3439–47. doi: 10.1007/s10067-020-05123-2 32424657

[29] Ucar AK Ozdede A Kayadibi Y Adaletli I Melikoglu M Fresko I. Increased arterial stiffness and accelerated atherosclerosis in takayasu arteritis. Semin Arthritis Rheum (2023) 60:152199. doi: 10.1016/j.semarthrit.2023.152199 37011578

[30] Cicco S Desantis V Vacca A Cazzato G Solimando AG Cirulli A. Cardiovascular risk in patients with takayasu arteritis directly correlates with diastolic dysfunction and inflammatory cell infiltration in the vessel wall: a clinical, ex vivo and *in vitro* analysis. Front Med (Lausanne) (2022) 9:863150. doi: 10.3389/fmed.2022.863150 35652080 PMC9149422

[31] D'Ascenzo F Presutti DG Picardi E Moretti C Omede P Sciuto F. Prevalence and non-invasive predictors of left main or three-vessel coronary disease: evidence from a collaborative international meta-analysis including 22 740 patients. Heart (2012) 98:914–9. doi: 10.1136/heartjnl-2011-301596 22626899

[32] Silveira LH. Cardiovascular manifestations of systemic vasculitides. Curr Rheumatol Rep (2020) 22:72. doi: 10.1007/s11926-020-00952-1 32856161

[33] Thuijs D Kappetein AP Serruys PW Mohr FW Morice MC Mack MJ. Percutaneous coronary intervention versus coronary artery bypass grafting in patients with three-vessel or left main coronary artery disease: 10-year follow-up of the multicentre randomised controlled SYNTAX trial. Lancet (2019) 394:1325–34. doi: 10.1016/S0140-6736(19)31997-X 31488373

[34] Ci W Zhao Y Bi T. Male Patients with takayasu arteritis and coronary artery involvement are prone to have serious coronary stenosis and high mortality. Curr Vasc Pharmacol (2022) 20:62–8. doi: 10.2174/1570161119666210720114939 34303330

[35] Wang H Zhang Y Shen Z Fang L Liu Z Zhang S. Comparing the effects of different management strategies on long-term outcomes for significant coronary stenosis in patients with takayasu arteritis. Int J Cardiol (2020) 306:1–7. doi: 10.1016/j.ijcard.2020.02.051 32115273

[36] Liu L Li J Gan T Yang Y Tian X. Isolated coronary arteritis in adults: a single-center experience from China. J Cardiovasc Transl Res (2023). doi: 10.1007/s12265-023-10388-4 37097590

[37] Zhou Y Feng Y Zhang W Li H Zhang K Wu Z. Physical exercise in managing takayasu arteritis patients complicated with cardiovascular diseases. Front Cardiovasc Med (2021) 8:603354. doi: 10.3389/fcvm.2021.603354 34055922 PMC8149735

