# Supplementary material for: Monocyte-to-high-density lipoprotein ratio as a predictor for patients with Takayasu arteritis and coronary involvement: a double-center, observational study
Source: Front Immunol. 2023 Jun 22;14:1120245. doi: 10.3389/fimmu.2023.1120245 (PMC10324657; doi:10.3389/fimmu.2023.1120245)
Supplement: Supplementary file 1 [file Table_1.docx]

| **Supplementary TABLE 1** Baseline characteristics of patients with and without LMD/3VD in TAK | | | | |  |
| --- | --- | --- | --- | --- | --- |
| Parameters | Without LMD/3VD  (n = 98) | With LMD/3VD  (n = 17) | *P-*value | |  |
| Age, years | 41.00 ± 12.46 | 43.71 ± 11.22 | 0.404 | |  |
| Male, n (%) | 11 (11.2) | 3 (17.6) | 0.729 | |  |
| Age at TAK onset, years | 33.39 ± 12.19 | 37.35 ± 11.89 | 0.217 | |  |
| TAK duration, months | 38.00 (12.00, 120.00) | 24.00 (10.00, 120.00) | 0.610 | |  |
| BMI, kg/m^2^ | 22.68 ± 3.26 | 24.91 ± 2.87 | **0.009** | |  |
| Symptoms, n (%) | |  |  | |  |
| Fever | | 5 (5.1) | 0 (0.0) | | 1.000 |
| Malaise | | 23 (23.5) | 3 (17.6) | | 0.829 |
| Weight loss | | 7 (7.1) | 3 (17.6) | | 0.341 |
| Arthralgia | | 11 (11.2) | 0 (0.0) | | 0.314 |
| Carotodynia | | 6 (6.1) | 0 (0.0) | | 0.590 |
| Diminished or absent pulse | | 16 (16.3) | 0 (0.0) | | 0.157 |
| Blood pressure inequality | | 12 (12.2) | 0 (0.0) | | 0.274 |
| Limb claudication | | 35 (35.7) | 2 (11.8) | | 0.051 |
| Chest pain | | 30 (30.6) | 10 (58.8) | | **0.024** |
| Dyspnea | | 34 (34.7) | 9 (52.9) | | 0.151 |
| Palpitations | | 11 (11.2) | 3 (17.6) | | 0.729 |
| Medical history, n (%) |  |  |  | |  |
| Hypertension | 44 (44.9) | 7 (41.2) | 0.776 | |  |
| Dyslipidemia | 28 (28.6) | 8 (47.1) | 0.129 | |  |
| Smoking | 10 (10.2) | 3 (17.6) | 0.631 | |  |
| Diabetes mellitus | 6 (6.1) | 1 (5.9) | 1.000 | |  |
| Family history of premature CAD | 7 (7.1) | 2 (11.8) | 0.868 | |  |
| Myocardial infarction | 4 (4.1) | 5 (29.4) | **0.002** | |  |
| Heart failure | 24 (24.5) | 7 (41.2) | 0.256 | |  |
| Lipid-lowering therapy at baseline, n (%) | 14 (14.3) | 5 (29.4) | 0.231 | |  |
| Numano classification, n (%) |  |  |  | |  |
| Type I | 16 (16.3) | 2 (11.8) | 0.907 | |  |
| Type IIa | 6 (6.1) | 1 (5.9) | 1.000 | |  |
| Type IIb | 15 (15.3) | 2 (11.8) | 0.992 | |  |
| Type III | 4 (4.1) | 1 (5.9) | 0.558 | |  |
| Type IV | 4 (4.1) | 1 (5.9) | 0.558 | |  |
| Type V | 33 (42.3) | 10 (58.8) | 0.215 | |  |
| Active disease of TAK, n (%) | 85 (86.7) | 14 (82.4) | 0.919 | |  |
| White blood cell, 10^9^/L | 6.35 (5.37, 7.43) | 7.00 (5.28, 8.13) | 0.741 | |  |
| Neutrophil, 10^9^/L | 3.83 (3.16, 5.04) | 3.96 (3.19, 5.79) | 0.587 | |  |
| Lymphocyte, 10^9^/L | 1.98 ± 0.57 | 1.96 ± 0.59 | 0.909 | |  |
| Neutrophil-to-lymphocyte ratio | 2.07 (1.66, 2.66) | 2.18 (1.57, 2.73) | 0.562 | |  |
| Monocyte, 10^9^/L | 0.35 (0.28, 0.45) | 0.35 (0.31, 0.47) | 0.410 | |  |
| eGFR, mL/min/1.73 m^2^ | 111.86 (98.48, 130.75) | 119.02 (101.76, 135.05) | 0.440 | |  |
| Triglyceride, mmol/L | 1.02 (0.73, 1.45) | 1.21 (1.03, 2.04) | **0.021** | |  |
| Total cholesterol, mmol/L | 4.29 ± 1.04 | 4.66 ± 1.63 | 0.377 | |  |
| LDL-C, mmol/L | 2.58 ± 0.87 | 3.06 ± 1.46 | 0.202 | |  |
| HDL-C, mmol/L | 1.11 (0.97, 1.42) | 0.92 (0.85, 1.04) | **0.001** | |  |
| Elevated ESR, n (%) | 38 (38.8) | 9 (56.3) | 0.188 | |  |
| Hs-CRP, mg/L | 1.82 (0.55, 10.18) | 5.19 (2.08, 26.20) | **0.022** | |  |
| MHR | 0.32 (0.22, 0.40) | 0.40 (0.33, 0.58) | **0.007** | |  |
| Normally distributed numerical variables (e.g., age, age at TAK onset, BMI, lymphocyte, total cholesterol, and LDL-C) were indicated as the mean ± standard deviation, and Student’s t-test was used for comparisons between groups. Non-normally distributed numerical variables (e.g., TAK duration, white blood cell, neutrophil, neutrophil-to-lymphocyte ratio, monocyte, eGFR, triglyceride, HDL-C, hs-CRP, and MHR) were indicated as the median (P25, P75), and a Mann–Whitney U test was used for comparisons between groups. Categorical data (e.g., sex, symptoms, medical history, lipid-lowering therapy at baseline, Numano classification, active disease of TAK, and elevated ESR) were indicated as n (%), and a Chi-square test was used for comparisons between groups. BMI, body mass index; CAD, coronary artery disease; eGFR, estimated glomerular filtration rate; ESR, erythrocyte sedimentation rate; HDL-C, high-density lipoprotein cholesterol; hs-CRP, high-sensitivity C-reactive protein; LDL-C, low-density lipoprotein cholesterol; LMD/3VD, left main disease and/or three-vessel disease; MHR, monocyte-to-high-density lipoprotein ratio; TAK, Takayasu arteritis. P-values <0.05 are shown in bold | | | | |  |
